# Racial equity in the fight against COVID-19: a qualitative study examining the importance of collecting race-based data in the Canadian context

**DOI:** 10.1186/s40794-021-00138-2

**Published:** 2021-06-10

**Authors:** Ranie Ahmed, Omer Jamal, Waleed Ishak, Kiran Nabi, Nida Mustafa

**Affiliations:** 1 Department of Health and Society, University of Toronto, 1265 Military Trail, Toronto, ON M1C 1A4 Canada; 2 Department of Biological Sciences, University of Toronto, 1265 Military Trail, Toronto, ON, M1C 1A4 Canada; 3 Department of Human Biology, University of Toronto, 100 St. George St, Toronto, ON M5S 3G3 Canada

**Keywords:** Racial equity, COVID-19, Black community, Social determinants of health, Racialized communities

## Abstract

**Background:**

A failure to ensure racial equity in response to the COVID-19 pandemic has caused Black communities in Canada to disproportionately be impacted. The aim of the current study was to determine the needs and concerns of Black communities in the Greater Toronto Area (GTA) and to highlight the importance of collecting race-based COVID-19 data early on to address these needs.

**Methods:**

Six qualitative interviews were conducted with local community health centre leaders who serve a high population of racialized communities within the GTA. Content analysis was used to extract the main themes and concerns raised during the interviews.

**Results:**

The findings from this study provide further evidence of the disproportionate impact COVID-19 has had on Black and other racialized communities. Difficulty self-isolating due to overcrowded housing, food insecurity, and less social support for seniors were concerns identified by community health leaders. Also, enhanced financial support for front-line workers, such as Personal Support Workers (PSWs), was an important concern raised. In order to lessen the impact of the pandemic on these communities, leaders noted the need for greater accessibility of testing centres in these areas and a greater investment in tailored health promotion approaches.

**Conclusions:**

Overall, our findings point to the importance of collecting race-based data to ensure an equitable response to the pandemic. The current “one size fits all” response is not effective for all individuals, especially Black communities. Not all populations have access to the same resources, nor do they live in the same conditions (Kantamneni, J Vocal Behav 119:103439, 2020). A deeper consideration of the social determinants of health are needed when implementing COVID-19 policies and responses. Also, a lack of attention to Black communities only continues to perpetuate the under-acknowledged issue of anti-Black racism prevalent in Canada.

## Background

COVID-19 is an infectious disease caused by the SARS-CoV-2 virus, targeting the respiratory and gastrointestinal tract of humans [1]. It is spread through respiratory droplets and airborne transmission, and manifests both asymptomatically and symptomatically within individuals [2]. Good hygiene, including hand sanitization, the use of facial masks/coverings and social distancing, are key protective measures identified by public health organizations, agencies and leaders across the globe.

Although the virus does not discriminate in terms of who it infects, research has shown that marginalized communities are at greater risks due to an inequitable distribution of resources, such as lack of government funding, housing, social support, and food insecurity [3]. Many marginalized populations are not able to adopt adequate preventative measures due to these conditions, which further increases their susceptibility to contract the virus [3]. In Canada, research shows that Black communities, in particular, are more likely to become sick and be hospitalized with COVID-19 compared to other ethnic groups [4]. For example, from May 20 to July 16 (2020), it was found that Black individuals made up 21% of the COVID-19 cases in Toronto (one of the most populated cities in the country), despite only making up 9% of the population [4]. These communities are also disproportionately affected by certain chronic conditions, such as HIV, diabetes, food insecurity, low-income, and unstable housing in Canada [5]. The effects of COVID-19 have, therefore, further exacerbated these already present health disparities.

Race-based data is the collection of population health outcomes stratified by racial groups [6]. Although some provinces in Canada, including Ontario, have started collecting this data, measures should have been taken earlier to ensure vulnerable communities are protected during this time. It has been suggested that the province should have gathered neighborhood based COVID-19 data in the early stages of the pandemic, or even prior, to identify communities that would be most impacted [6]. This data could have guided and informed equitable response efforts, such as providing additional funding and safety resources (e.g. face masks, sanitizers) to those communities. Also, the government could have taken preventive measures such as establishing priority food drives, pop-up testing clinics, and resource donation drives (e.g. hand sanitizers, masks, personal protective equipment, etc.) in the locations that have the highest prevalence of cases, which are marginalized communities.

Race-specific data collected early on could have guided a more equitable response to the pandemic as we would have clearly seen the intersection of race, income, location of residence, education, occupation/employment and resource availability. If collected earlier, these variables could have helped public health officials understand the challenges, barriers and living conditions of different racial and marginalized populations and the impact the pandemic could have on these communities. In times of crisis, the inequitable experiences of marginalized populations need to be identified and addressed at the beginning, as these groups disproportionately bear a greater burden of suffering [7]. Hence, the goal of this project was to demonstrate the importance of collecting race-based COVID-19 data to ensure an equitable response to the current pandemic, in the Canadian context.

## Materials and methods

### Qualitative study design

Qualitative research is a process that involves collecting and analyzing non-numerical data to achieve an improved understanding of a specific concept, opinion, or experience [8]. It provides researchers with personal accounts and experiences on various topics, and a deeper understanding of a problem to help generate new ideas for research. Unlike quantitative research, which uses statistics and numerical data, qualitative research allows researchers to further understand population health outcomes based on conversations, narratives, and discussions with those who have lived experiences of an issue, or those who work closely with members within a community.

In order to understand the issue of racial equity in this matter, we decided to take a qualitative approach. This issue cannot be examined through health statistics because the lived experiences of the target populations through community-based research is required. This provides a powerful means to examine concerns and challenges which cannot be quantified through quantitative measures, and so our project utilized this approach.

### Data collection method

After obtaining ethical approval from a University’s Research Ethics Board, six qualitative interviews were conducted with five executive directors of community health centre leaders across the GTA and one University professor, between July 2020 to August 2020. Interviews were conducted through zoom, an online communication platform, due to COVID-19 policies and guidelines.

Each interview was between 45 min to 1 h. Community leaders were asked about issues and concerns within their communities in regards to the pandemic; for example, “what resources are currently needed that the community does not have access to?”, “how important is it that our leaders understand that a one-size fits all approach is not an effective solution this pandemic?”. Also, community leaders were asked for their thoughts on the importance and need for collecting race-based data in the GTA. It was important to interview these community health leaders as they deal directly, communicate and interact with members of the Black community. Individuals of the Black community often rely on these health centres for aid and advice regarding their personal health. Since the communities are tightly knit, the lived experiences of its members are truly understood by the community health leaders who are involved in voicing the community’s concerns to the government. Hence, in our study we aimed to target these community leaders as they were able to provide us with valuable insight into the lived experience of the members within their community.

Once interviews were complete, they were transcribed verbatim, followed by the extraction of main themes through the use of Content Analysis [9]. This systematic method identifies important and emerging thematic findings that relate to the overall research focus/question.

## Results

The findings below identify the main concerns which arose among Black communities during the pandemic. In particular, community leaders discussed the importance of mobile testing and the role of working conditions in spreading the virus among marginalized groups. They also identified the role of anti-Black racism in the pandemic response and the importance of equitable health policies. Future lessons/approaches the government should take to implement equitable public health measures that are tailored to the lived experiences of Black communities, were also highlighted.

### Common concerns of the community

A common concern emerging from the interviews was a reluctance among individuals from Black communities to get tested for the virus due to misunderstandings of how painful the test would be. Also, there was a fear of increased contact with individuals who have tested positive at the testing centre. This made individuals from these communities less likely to get tested for the virus. In addition, individuals were hesitant to get tested due to the possibility of a positive test, leading them to worry about taking time off work. This would mean they would need to find other sources of income to support their families (i.e. to pay rent, buy groceries).

Many members of these communities also lost jobs and were not eligible for the Canadian Emergency Response Benefit (CERB). This led to other concerns such as the affordability of food and personal protective equipment (PPE). As many members from these communities have a lower socio-economic status, the loss of employment further exacerbated income inequality.

These concerns provide clarity on the negative effects of COVID-19 on Black communities, and the impact of this on the communities’ physical and mental health.

### Mobile testing

Mobile testing became available in non-racialized GTA neighborhoods (which were socially less burdened) weeks before regions that were most affected by COVID-19. In particular, those communities with inadequate housing and poor access to healthcare were delayed in receiving such resources. Communities with greater populations of racialized individuals suffered the most during the pandemic because there was no prior knowledge of the needs of these communities or what health concerns were already present. Community leaders suggested that differences in resource allocation between certain communities’ points to systemic discrimination, neglect, and a lack of prioritization of racialized communities which are in need the most.

### Working conditions

A disproportionate number of Black female personal support workers (PSW) were employed in nursing homes which were most affected during the pandemic. This increased their chances of contracting the virus themselves, as well as becoming carriers for their family members.

Also, community leaders indicated a correlation between low-income jobs and the risk of contracting COVID-19, in these communities specifically. As one leader highlighted, “a lot of racialized and marginalized communities live in low-income conditions, where their employment situation is precarious to an extent where they aren’t able to access financial assistance” (Executive Director, Community Health Centre in Scarborough, Toronto). Thus, to make ends meet, some Black individuals have to work multiple jobs to receive adequate income.

### Psychological effects

The psychological effects of COVID-19 are expected to have a significant impact on populations across the country, especially those of marginalized communities who face a greater burden. For example, many individuals from these regions must work throughout the pandemic as they do not have the option of staying home. Often times, these individuals are living with vulnerable family members (e.g. seniors; those with chronic conditions), and therefore, have to send them to other family member’s houses to reduce the spread of COVID-19. This adds to the isolation, loneliness, and depression these communities face. The psychological effects are cumulating; therefore, resources need to be tailored and made accessible in these communities. One community leader noted that, “there are accessibility issues to mental health support in these communities already” (Executive Director, Community Health Centre in Scarborough, Toronto). Thus, efforts need to be made to increase access to mental health services in Black communities.

### Anti-black racism

Community leaders highlighted that earlier measures should have been implemented to avoid the high percentage of COVID-19 cases among Black people and other people of colour. Racial inequities faced by communities in the healthcare system continues to be a long-standing concern even prior to the current pandemic. Health officials, therefore, need to acknowledge and prioritize the health of minority populations through the implementation of tangible policies that will produce meaningful change.

### Policies

It was also noted that health officials need to review policies and create new ways to respond to COVID-19, which target anti-Black racism, specifically. Similar to the importance of collecting race-based data, it is important for policies to reflect the lived experiences of racialized populations in a real, structural, and meaningful way. Community leaders identified that many institutions already have such policies in place; however, these policies do not always translate into practice.

### Lessons/future approaches

The provincial delay to collect race-based data is an important lesson to keep in mind, according to community leaders. Black and other racialized communities are impacted by COVID-19 and (other chronic conditions) significantly more than other populations. The inequitable disparities that already exist in these communities (in terms of housing, income, food security), perpetuate these problems even further.

As one leader notes, “the common denominator is anti-Black racism and a system that is generating all of these problems. With the COVID-19 pandemic and what has transpired after the Black Lives Matter (BLM) movement [and recent attention given to the BLM in 2020], we as a society have been given a good opportunity to think differently about race and health, and to bring about change” (Executive Director, Community Health Centre in Scarborough, Toronto). Public health officials, governments, and the overall population, can, and should shift the needle towards equity and fairness in health, and overall quality of life.

An important point raised by those in the study was the importance of considering how different communities will access health services when the virus vaccine is ready to distribute, in order to ensure effective usage. Making vaccinations available where community members are most comfortable visiting will be important to consider. Public health officials also need to raise awareness within these communities and promote community engagement to overcome misinformation and myths about vaccinations. In addition, Black communities particularly have a difficult time trusting the health system, since health services, including clinical trials and vaccines, have previously been used to further racialize, marginalize, and kill members of the Black community. Therefore, community leaders encourage and support an equitable deployment plan for vaccination.

Also, community leaders mentioned that they would like to see relationship and community building within Black communities to encourage the use of testing and mobile testing units. This will ensure trust is built and members of the community feel comfortable taking the vaccination. Normally, vaccinations are given through institutions and hospitals; however, this may not be accessible to all Black community members. Therefore, there needs to be a shift in planning to ensure a more equitable approach to intervention and prevention.

## Discussion

The Canadian federal, provincial, and municipal government’s general response to the pandemic has been commendable, as we saw a flattening of COVID-19 cases early on. Unfortunately, there still remains gaps in the lack of community-specific responses that are racially and socially equitable. Not all individuals should be treated the same way, as people do not have the same opportunities, privileges, and access to health care [3]. Collecting race-based data prior to the pandemic, would have made this more evident. In that case, more community-specific responses could have been implemented, which could have led to a significant reduction in the virus cases we see today. However, since there is not enough detailed race-based data available that clearly shows the impact of the pandemic on Black communities across the GTA, the current issues remain. For example, current COVID-19 policies and responses are not tailored to meet the needs and concerns of Black communities, as noted earlier in our findings. The Canadian government’s neglect toward these communities not only prevents an equitable response, but worsens the health of these communities. Moving forward, there should be specific plans and strategies put in place for Black communities, as well as other minority groups such as Indigenous peoples and those living in low-income communities, which ensure better health outcomes for all.

In June 2020, the City of Toronto declared anti-Black racism a public health crisis [10]. The question remains on what our governmental systems and structures will do to translate this declaration into the pandemic response. Public health officials need to prepare and implement an equitable response, especially knowing that 80% of COVID-19 cases are among Black people and those of colour [4]. The large proportion of Black community members who have contracted the virus further shows the systemic racism which contributes to the current inequity. If race-based data was collected early on, the high percentages could have been avoided with greater resources allocated to the communities which need them the most. The anti-Black racism experienced by Black individuals also adds to the growing distrust from the community towards the health care system. This will need to be rectified by building trust between Black communities and the healthcare system through the establishment of meaningful partnerships. This can be done by creating policies that acknowledge and create culturally sensitive systems, as well as targeted resources, including mental health support. Furthermore, efforts should be made to recognize the generational trauma Black communities have faced at the hands of the healthcare system.

The collection of race-based data across the country is, therefore, extremely necessary to ensure this response is equitable. Currently, public health officials have begun collecting this data, however, they need to be better prepared to desegregate the data to identify which regions are most disproportionately impacted. If action is taken now, there is promise for equitable changes within our health and social systems.

### Limitations

This study has potential limitations. Firstly, the sample size for the interviews was limited. Due to the timeline of the study and COVID-19 restrictions, directly reaching Black community members during this time was not feasible. We were, therefore, not able to directly interview Black community members who are not professionals due to restrictions that were in place during our project (i.e. 4 month timeline). Recruitment of these individuals would have taken significantly longer, especially due to the second-wave of the pandemic. However, we decided to optimize our findings by reaching out to the community health centre leaders who represent these marginalized populations. We strongly believe that by interviewing the community health centre leaders within pre-dominantly Black communities, we were able to capture broad needs and concerns of members within these communities. This is because the leaders are constantly involved and engaged with the community and their needs, and have a strong understanding of their lived experiences. Secondly, we were unable to target a larger demographic in this study, as only the Black communities of the GTA were included. Future studies should consider exploring this issue beyond the GTA, to avoid selection bias.

## Conclusion

The COVID-19 pandemic has and continues to disproportionately impact Black communities, which highlights the importance of collecting race-based data to ensure an equitable response. The collection of this data will inform health officials on the needs of vulnerable communities, followed by effective resource allocation. Collecting race-based data is one step towards addressing the health inequities faced by Black communities in Canada.

## Abbreviations

GTA: Greater Toronto Area
COVID- 19: Coronavirus disease 2019
PSWs: Personal Support Workers
SARS-CoV-2: Severe acute respiratory syndrome, coronavirus 2
BME: Black and minority ethnic communities
CERB: Canadian Emergency Response Benefit
PPE: Personal protective equipment
